# Case Report: IL2RA (CD25) deficiency: first reported cases in Morocco

**DOI:** 10.3389/fimmu.2026.1753561

**Published:** 2026-01-30

**Authors:** Ahamada Elamine, Ibtihal Benhsaien, Abderrahmane Errami, Yousra Bellarhrib, Zahra Aadam, Mohammed Fahi, Ahmed Aziz Bousfiha, Jalila El Bakkouri, Fatima Ailal

**Affiliations:** 1 Laboratory of Clinical Immunology, Infection and Autoimmunity (LICIA), Faculty of Medicine and Pharmacy, Hassan II University, Casablanca, Morocco; 2 Department of Pediatric Infectious Diseases and Clinical Immunology, Mother-Child Hospital Abderrahim El Harouchi, Ibn Rochd University Hospital Center, Casablanca, Morocco; 3 Immuno-serology Laboratory, Ibn Rochd University Hospital Center, Casablanca, Morocco; 4 Immunopathology-Immunotherapy-Immunomonitoring Laboratory, Faculty of Medicine, Mohammed VI University of Health Sciences (UM6SS), Casablanca, Morocco

**Keywords:** autoimmunity, CD25 deficiency, IL2RA deficiency, immune dysregulation, inborn errors of immunity, IPEX-like syndrome, Morocco, regulatory T cells

## Abstract

CD25, the α-chain of the interleukin-2 receptor (IL2RA), is a key component of the IL-2 pathway and is essential for the development and stability of regulatory T cells. Loss-of-function variants in IL2RA cause a very rare autosomal recessive disorder marked by early-onset autoimmunity and recurrent infections with an IPEX-like presentation. We report the first two molecularly confirmed cases of IL2RA (CD25) deficiency in Morocco, each carrying a distinct homozygous mutation. Both patients were born to first-cousin parents and presented in early childhood with recurrent respiratory and gastrointestinal infections, severe failure to thrive, chronic diarrhea with celiac-like enteropathy, and autoimmune manifestations including autoimmune hepatitis, dermatitis, and, in one case, autoimmune thyroiditis. Lymphocyte subset counts and immunoglobulin levels were within or above age-appropriate ranges, but flow cytometry showed a complete absence of CD25 expression on CD4^+^ T cells in both children, whereas relatives displayed intermediate levels compatible with carrier status. Targeted next-generation sequencing identified two novel IL2RA variants: a splice-site mutation (c.65-2A>G) and a multi-exon deletion (c.557_795-1625del), both leading to loss of functional CD25. Both variants were absent from population databases and classified as likely pathogenic or pathogenic according to ACMG criteria. These two cases expand the mutational and geographic spectrum of IL2RA deficiency and highlight the importance of considering this diagnosis in infants from consanguineous families who present with unexplained polyautoimmunity and recurrent infections. Simple flow cytometric assessment of CD25 on T cells is a valuable screening tool, and early genetic confirmation is crucial to guide timely hematopoietic stem cell transplantation and genetic counselling.

## 1 Introduction

Interleukin-2 (IL-2) is a central cytokine in adaptive immunity, essential for T-cell activation, proliferation, and the maintenance of immune homeostasis (1, 2). IL-2 signaling is mediated through the interleukin-2 receptor (IL-2R) complex, which is composed of three subunits: IL-2Rα (CD25), IL-2Rβ (CD122), and the common γ chain (CD132) (3). The IL-2Rβ and γc subunits are responsible for signal transduction and are shared with other cytokine receptors, including those for IL-4, IL-7, IL-9, IL-15, and IL-21 (4). In contrast, IL-2Rα (CD25) is unique to the IL-2 receptor complex and has no signaling capability on its own. Its principal function is to enhance receptor affinity for IL-2, allowing efficient ligand capture even at low cytokine concentrations (5). In conventional T lymphocytes, CD25 expression is transiently induced upon activation and generally remains of low intensity, in contrast to the constitutive and high-level expression observed on regulatory T lymphocytes (Tregs), where it plays a pivotal role in mediating IL-2-driven STAT5 activation. This signaling is essential for the development, stability, and function of Tregs, and thereby critical for maintaining self-tolerance and immune homeostasis (5, 6). Although the impact of CD25 deficiency has been predominantly described in T lymphocytes, accumulating evidence indicates that certain NK cell populations particularly the CD56^bright^ subset constitutively express CD25 and can assemble a high-affinity IL-2 receptor, enabling them to respond to low concentrations of IL-2. For instance, Caldirola et al. reported impaired NK cell maturation and function in a patient carrying an IL2RA mutation, highlighting the importance of the IL-2/CD25 axis in NK cell biology (7). In addition, Hirakawa et al. demonstrated that CD56^bright^ NK cells are activated in response to low-dose IL-2 through high-affinity IL-2 receptors, further supporting the contribution of CD25 to NK cell IL-2 sensitivity (8).

CD25 deficiency is a very rare autosomal recessive monogenic immunodeficiency marked by profound immune dysregulation (9). Clinical manifestations usually begin in early infancy, with patients presenting with recurrent bacterial, viral, and fungal infections, failure to thrive, and autoimmune manifestations such as enteropathy, cytopenias, eczema, and endocrinopathies (10–18). The first human case was reported in 1997 and involved a child with severe T-cell dysfunction and diffuse lymphocytic organ infiltration (10). A decade later, it was shown that CD25 deficiency can mimic IPEX (Immunodysregulation, Polyendocrinopathy, Enteropathy, X-linked) syndrome, even in the absence of FOXP3 mutations (11). In such cases, defective IL-10 production by CD4^+^ T cells illustrates the failure of immune regulation (11).

Over the past two decades, fewer than twenty patients with *IL2RA* mutations have been reported worldwide (10–18). These cases confirmed that loss of CD25 expression results in defective IL-2 signaling and a breakdown of peripheral immune tolerance. Moreover, beyond these rare loss-of-function cases, common *IL2RA* polymorphisms have been linked to susceptibility to several autoimmune diseases in the general population, including type 1 diabetes, multiple sclerosis, and juvenile idiopathic arthritis (19–23). Such observations underscore the broader importance of the IL-2/CD25 axis in immune regulation.

**Figure 1:**
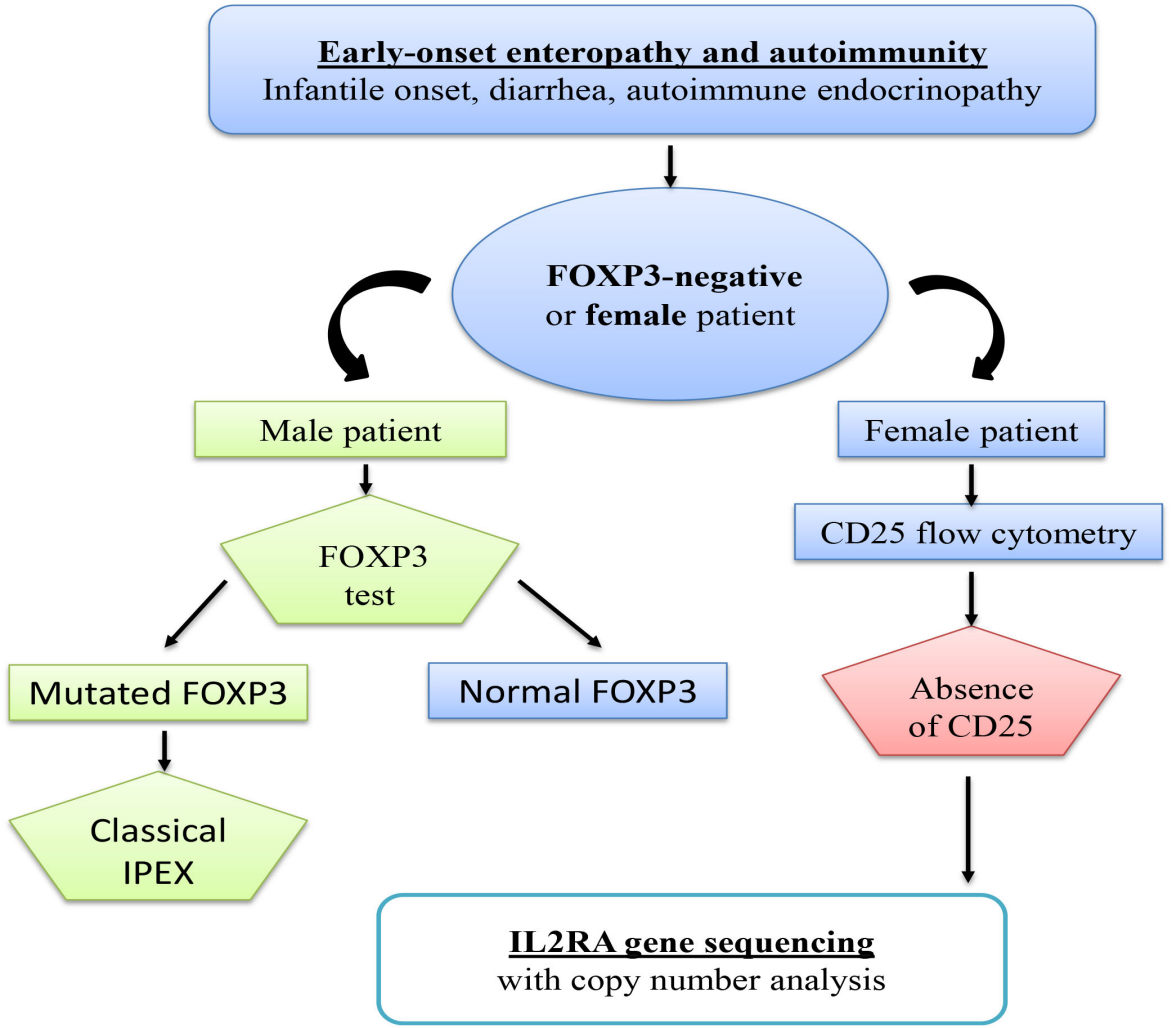
Diagnostic approach to IPEX-like syndromes.

Here we present the first two confirmed cases of IL2RA (CD25) deficiency in Morocco. We describe their clinical phenotypes, immunological profiles, and the discovery of two novel biallelic IL2RA mutations. To our knowledge, no molecularly confirmed cases of CD25 deficiency have previously been reported from Morocco or North Africa. These findings expand the known genotypic spectrum of IL2RA mutations and reinforce the diagnostic importance of CD25 staining by flow cytometry in patients with IPEX-like syndromes (**Figure 1**). Given the high rate of consanguinity in the region and the availability of curative hematopoietic stem cell transplantation, early recognition of this rare but life-threatening disorder is crucial.

## 2 Methods

### 2.1 Patients and clinical evaluation

The two patients described in this study were evaluated and followed at the Department of Pediatric Infectious Diseases and Clinical Immunology, Mother-Child Hospital Abderrahim El Harouchi, University Hospital Ibn Rochd, Casablanca, Morocco. Detailed clinical assessments were performed, including medical history, physical examination, and relevant laboratory. Family pedigrees were obtained through direct interviews with parents.

### 2.2 Immunophenotyping by flow cytometry

CD25 surface expression on CD4^+^ T cells was assessed by flow cytometry. Peripheral whole blood samples were collected in EDTA tubes and processed within 24 hours. Red blood cells were lysed using an ammonium chloride-based lysing buffer. Leukocytes were stained with fluorochrome-conjugated monoclonal antibodies, including anti-CD45-V500, anti-CD3-PerCP-Cy5.5, anti-CD4-V450, and anti-CD25-PE (BD Biosciences). After incubation, samples were acquired on a FACSLyric™ flow cytometer (BD Biosciences). Standard lymphocyte subset analysis was also performed using panels of monoclonal antibodies targeting CD3, CD4, CD8, CD19, and CD16/56. Results were interpreted using age-matched reference ranges.

### 2.3 Genetic analysis

Genomic DNA was extracted from peripheral blood leukocytes of the patients. Targeted next-generation sequencing (NGS) was performed using a custom gene panel encompassing known primary immunodeficiency genes (including *IL2RA*). Sequencing libraries were prepared by hybridization capture and sequenced on an Illumina platform. Sequence reads were aligned to the human reference genome (GRCh37) and variants were called and annotated following standard guidelines of the Human Genome Variation Society (HGVS). The population frequency of each candidate variant was checked using the GeneBe platform (https://genebe.net/). In silico prediction tools were employed to evaluate variant pathogenicity: for the splice-site mutation, we used splice site prediction algorithms (ADA and RF score from dbscSNV, among others) to assess impact on splicing; for both variants, we used a deleteriousness meta-score (BayesDel) and Combined Annotation Dependent Depletion (CADD) scoring to gauge the likelihood of pathogenic effect. The variants were interpreted according to ACMG criteria. Additionally, we utilized the PopViz (https://hgidsoft.rockefeller.edu/PopViz/) and Ensembl (https://www.ensembl.org/) databases to visualize the position of the mutations in the context of variant deleteriousness and population frequency.

### 2.4 Ethical considerations

The study was conducted in accordance with the ethical principles of the Declaration of Helsinki. Written informed consent was obtained from the legal guardians of both patients for clinical investigations, genetic testing, and the publication of anonymized data. Ethical approval was granted by the Ethics Committee of the Faculty of Medicine and Pharmacy, Hassan II University of Casablanca (approval number 06/2023).

## 3 Results

### 3.1 Clinical presentation

#### 3.1.1 Case 1 (patient 1)

**Figure 2:**
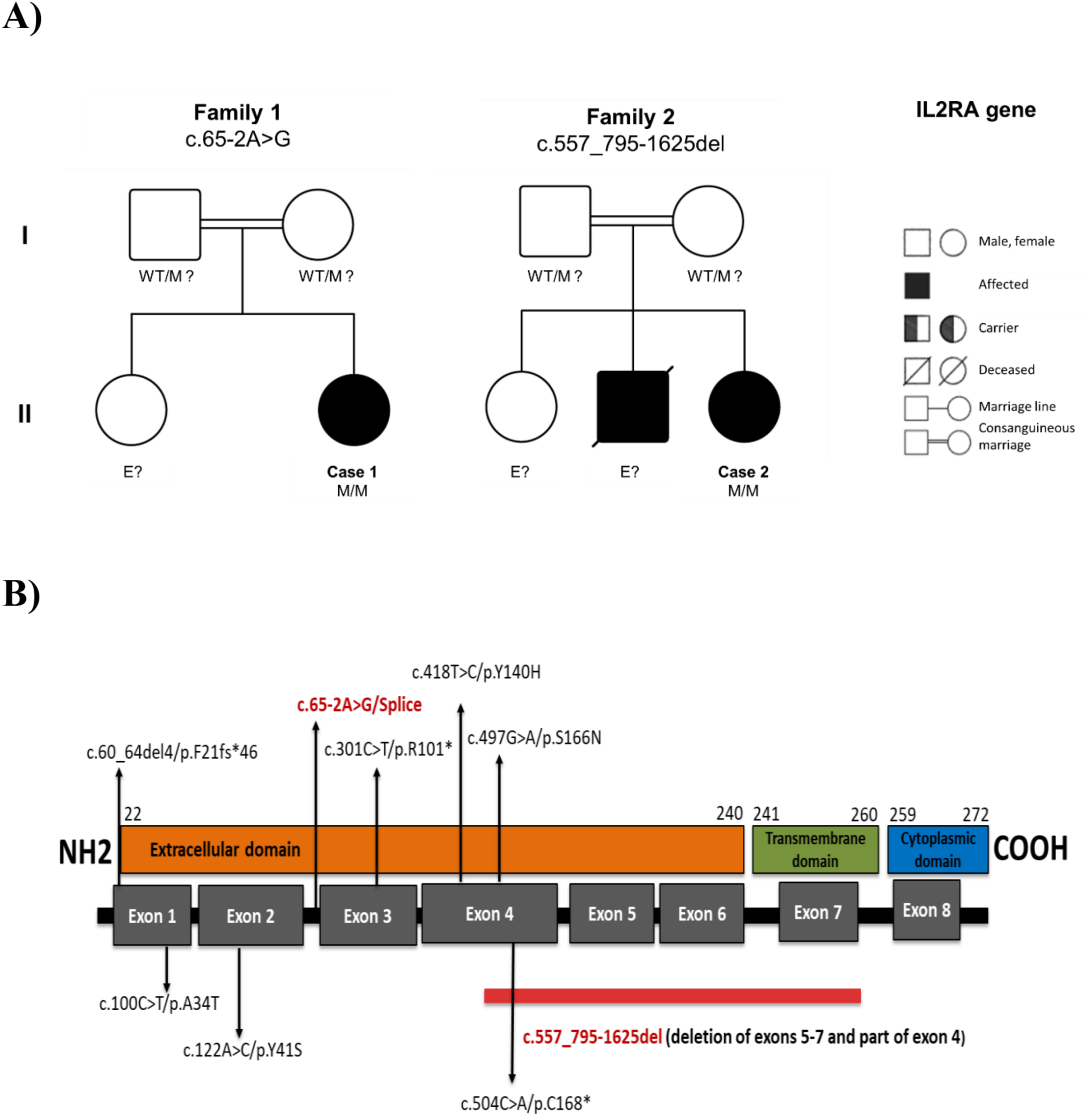
**(A)** Pedigrees of two unrelated Moroccan families with autosomal recessive IL2RA deficiency. Case 1 and Case 2 were both born to first-degree consanguineous parents. Affected individuals (black-filled symbols) are homozygous for distinct IL2RA mutations: c.65-2A>G in Family 1 and c.557_795-1625del in Family 2. The diagonally slashed square indicates a deceased sibling with a similar clinical phenotype, though not genetically tested. Parents are presumed heterozygous carriers (WT/M)?. Individuals labeled E? have unknown genetic status. WT: wild-type allele; M: mutant allele. Squares denote males; circles denote females. Double horizontal lines represent consanguineous unions. **(B)** Schematic representation of the IL-2Rα (CD25) protein structure with corresponding exon organization, showing the location of the mutations identified in our patients (highlighted in red) relative to previously reported IL2RA mutations (shown in black). The red horizontal bar indicates the multi-exon genomic deletion spanning exons 4–7 identified in Patient 2.

The first patient was a 3-year-old girl born to first-degree consanguineous parents (**Figure 2A**). She was admitted to our unit for febrile respiratory distress, with a history of recurrent infections and failure to thrive. Since the age of 12 months, she had experienced frequent febrile episodes (about three per month), two to three diarrheal episodes per month, and repeated respiratory tract infections, including several pneumonias requiring hospitalization. Her medical history was also marked by refractory anemia and hepatobiliary involvement with episodes of cholestatic jaundice. At 3 years of age, she developed autoimmune gastrointestinal and hepatic manifestations: endoscopy performed for persistent abdominal symptoms revealed petechial gastritis with fold hypertrophy, and anti–tissue transglutaminase antibodies were strongly positive, confirming celiac disease (autoimmune enteropathy). She was also diagnosed with autoimmune hepatitis. A skin biopsy performed for chronic dermatitis showed features consistent with eczema. On examination, she had hepatomegaly, splenomegaly, and generalized lymphadenopathy (**Table 1**).

**Table 1:** Clinical characteristics of the two Moroccan patients with IL-2Rα deficiency.

Feature	Case 1 (patient 1)	Case 2 (patient 2)
Age at last evaluation	3 years	8 years 4 months
Sex	Female	Female
Consanguinity	Yes (first cousins)	Yes (first cousins)
Recurrent infections	Frequent respiratory infections, multiple pneumonias, dysentery; persistent viral infections	Chronic respiratory infections and diarrheal illness
Failure to thrive	Yes	Yes
Autoimmune manifestations	Autoimmune enteropathy (celiac disease), autoimmune hepatitis, eczema	Autoimmune enteropathy (celiac disease), hypothyroidism, dermatitis (eczema)
Family history	No similar cases	Brother died at 4.5 years with similar illness

Laboratory investigations demonstrated moderate microcytic anemia (hemoglobin 9.3 g/dL; MCV 76.8 fL) and mild leukocytosis (10.98×10³/µL) with neutrophils 4.60×10³/µL and lymphocytes 4.86×10³/µL (**Table 2**). Platelets were slightly elevated (409×10³/µL). HIV serology was negative. Lymphocyte subset immunophenotyping revealed normal absolute T-, B-, and NK-cell counts for her age: CD3^+^ 3103/µL, CD4^+^ 1861/µL, CD8^+^ 881/µL, CD19^+^ 1221/µL, and CD16^+^/CD56^+^ 308/µL. Serum immunoglobulin testing showed marked hypergammaglobulinemia (IgG 33.56 g/L), while IgA (2.47 g/L), IgM (0.52 g/L), and IgE (50 IU/mL) were within normal ranges (**Table 2**).

**Table 2:** Laboratory and immunological findings of two Moroccan patients with IL-2Rα deficiency.

Parameter	Case 1 (3 years)	Reference range*	Case 2 (8 years)	Reference range*
WBC (×10³/µL)	10.98 ↑	5.0–10.1	17.0 ↑	4.0–9.2
Neutrophils (×10³/µL)	4.60	1.41–5.84	2.10	1.41–5.84
Lymphocytes (×10³/µL)	4.86	2.13–7.05	13.35 ↑	1.41–3.65
Hemoglobin (g/dL)	9.3 ↓	10.6–13.5	4.3 ↓	11.7–14.0
Platelets (×10³/µL)	409	222–444	443	222–444
IgG (g/L)	33.56 ↑	3.4–6.2	21, 02 ↑	5.8–10.8
IgA (g/L)	2.47 ↑	0.33–1.22	~0.7	0.49–1.57
IgM (g/L)	0.52	0.48–1.43	~1.0	0.54–1.53
IgE (IU/mL)	50	0–60	45	0–90
CD3^+^ T cells (×10³/µL)	3.10	1.40–3.70	3.63 ↑	1.20–2.60
CD4^+^ T cells (×10³/µL)	1.86	0.70–2.20	1.91 ↑	0.65–1.50
CD8^+^ T cells (×10³/µL)	0.88	0.49–1.30	1.56 ↑	0.37–1.10
CD19^+^ B cells (×10³/µL)	1.22	0.39–1.40	0.60	0.27–0.86
NK (CD16^+^/56^+^) cells (×10³/µL)	0.31	0.13–0.72	0.70 ↑	0.10–0.48
CD4^+^CD25^+^ T cells (%)	0.16 ↓	~20–30	0.00 ↓	~20–30
IL2RA variant	c.65-2A>G	—	c.557_795-1625del	—
ACMG classification	Likely pathogenic (PVS1, PM2, PP3)	—	Pathogenic (PVS1, PM2)	—

*Reference ranges: hematology values were adapted from CALIPER pediatric intervals (Bohn et al., 2023) (24). Immunoglobulin and lymphocyte subset ranges were based on the PID Phenotypical Diagnosis App (Jeddane et al., 2017) and Shearer et al., 2003;112:973–80 (25, 26). WBC, white blood cells; NK, natural killer cells. Symbols: ↑, above reference range; ↓, below reference range. ACMG, American College of Medical Genetics and Genomics variant classification system (PVS1, very strong pathogenic evidence; PM, moderate; PP, supporting).

The patient is currently receiving monthly intravenous immunoglobulin replacement therapy, along with antibacterial prophylaxis. She is maintained on a gluten-free diet and immunosuppressive treatment. Despite this management, she continues to experience intermittent episodes of lymphoproliferation, characterized by transient peripheral lymphadenopathy.

#### 3.1.2 Case 2 (patient 2)

The second patient was an 8-year-old girl, also born to first-cousin parents from a different family (**Figure 2A**). Her symptoms began in early infancy (**Table 1**). At 4 months of age, she was hospitalized for persistent unexplained fever, and by 5 months she had developed chronic diarrhea. Throughout infancy, she exhibited pronounced growth retardation and delayed motor development. At 2 years, evaluation for persistent diarrhea and failure to thrive revealed malabsorption with villous atrophy on intestinal biopsy. This was initially managed as celiac disease; her anti-transglutaminase antibody titers were exceedingly high (>300 IU/mL). Despite a gluten-free diet, her gastrointestinal symptoms persisted, raising suspicion of a more complex autoimmune enteropathy. She also had refractory iron-deficiency anemia, hepatosplenomegaly, and persistent lymphadenopathy. A liver biopsy performed due to prolonged hepatomegaly showed evidence of steatohepatitis. Around the age of 3, she developed signs of thyroid autoimmunity: cervical ultrasound revealed features of chronic thyroiditis, and she presented with anterior neck swelling. Thyroid function tests confirmed primary hypothyroidism (free T4 ~0.5 µg/dL, TSH >100 µIU/mL) with markedly elevated anti-thyroperoxidase antibodies (>600 IU/mL), consistent with autoimmune thyroiditis. She was started on thyroxine replacement therapy. She also had recurrent episodes of wheezing and dyspnea and chronic xerotic dermatitis with eczema-like features. Taken together, her clinical picture was consistent with early-onset immune dysregulation resembling an IPEX-like phenotype.

On examination, she had stunted growth and signs of chronic systemic illness. Laboratory testing revealed severe microcytic hypochromic anemia (hemoglobin 4.3 g/dL), leukocytosis (17.0×10³/µL), marked absolute lymphocytosis (13.35×10³/µL), and relative neutropenia (2.10×10³/µL). Platelets were elevated (443×10³/µL). HIV serology was negative. Lymphocyte subset immunophenotyping showed normal B-cell counts, CD19^+^ 605/µL, whereas T and NK cells were increased for her age: CD3^+^ 3632/µL, CD4^+^ 1917/µL, CD8^+^ 1564/µL and CD16^+^/CD56^+^ 706/µL (**Table 2**). Serum immunoglobulin testing showed marked hypergammaglobulinemia (IgG 21, 02 g/L). The other immunoglobulins were also within age-appropriate ranges (IgA ~0.7 g/L; IgM ~1.0 g/L). The therapeutic management was based on monthly intravenous immunoglobulin replacement therapy, combined with antibacterial prophylaxis, a gluten-free diet, and immunosuppressive treatment. Thyroid hormone replacement therapy with levothyroxine (4 mg/kg/day) was initiated. To date, the clinical course is characterized by stabilization of clinical manifestations. Her family history was notable for a deceased older brother who died at 4½ years after a similar clinical course characterized by recurrent respiratory infections, severe growth failure, autoimmune hepatitis, and enteropathy (**Figure 2A**). Although no genetic testing was performed at the time, his presentation strongly suggests he may have been affected by the same underlying immunological disorder.

### 3.2 CD25 surface expression

**Figure 3:**
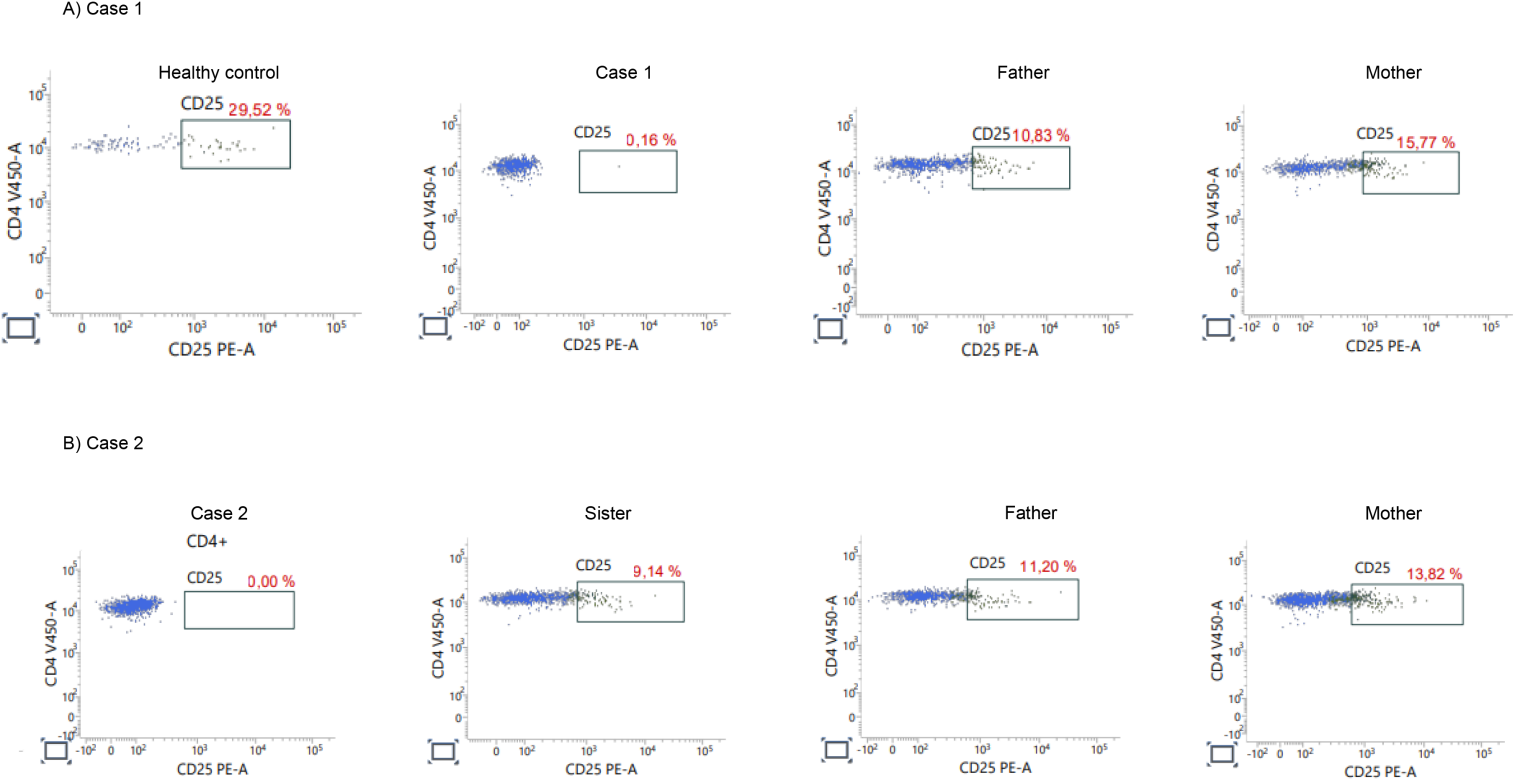
Flow cytometry analysis of CD25 expression on CD4^+^ T cells in patients and family members. Flow cytometric analysis of CD25 (IL-2Rα) expression on CD4^+^ T cells in Case 1 and Case 2, their relatives, and a healthy control. **(A)** Case 1: CD25^+^CD4^+^ T cells were nearly absent in the patient (0.16%) compared to a healthy control (29.52%). Both parents showed reduced CD25 expression (father: 10.83%; mother: 15.77%), consistent with presumed heterozygous carrier status. **(B)** Case 2: CD25^+^CD4^+^ T cells were undetectable in the patient (0.00%). Her sister and parents displayed intermediate CD25 expression levels (sister: 9.14%; father: 11.20%; mother: 13.82%), also supporting heterozygous status.

Given the autoimmune manifestations and lymphoproliferation observed in both cases, immunophenotyping focused on CD25 expression. Flow cytometric analysis of whole blood lymphocytes showed a complete absence of CD25 expression on CD4^+^ T cells in both patients. In healthy controls, approximately 20–30% of circulating CD4^+^ T cells express CD25. In Case 1, only 0.16% of CD4^+^ T cells expressed CD25, and in Case 2 the proportion was 0.00%, confirming a total lack of detectable CD25 on the cell surface (**Figure 2**). In contrast, the parents from both families displayed intermediate CD25 expression, with levels ranging from 9–15% of CD4^+^ T cells, clearly reduced compared with controls but higher than in the affected children, consistent with a heterozygous carrier status (**Figure 3**).

### 3.3 Genetic findings

Based on the absent CD25 expression, genetic analysis of IL2RA was performed. Targeted next-generation sequencing (NGS) of a primary immunodeficiency gene panel identified homozygous IL2RA variants in both patients, consistent with an autosomal recessive defect of the IL-2 receptor α chain (CD25). No additional variants clearly compatible with the clinical phenotype were detected in other genes on the panel.

**Figure 4:**
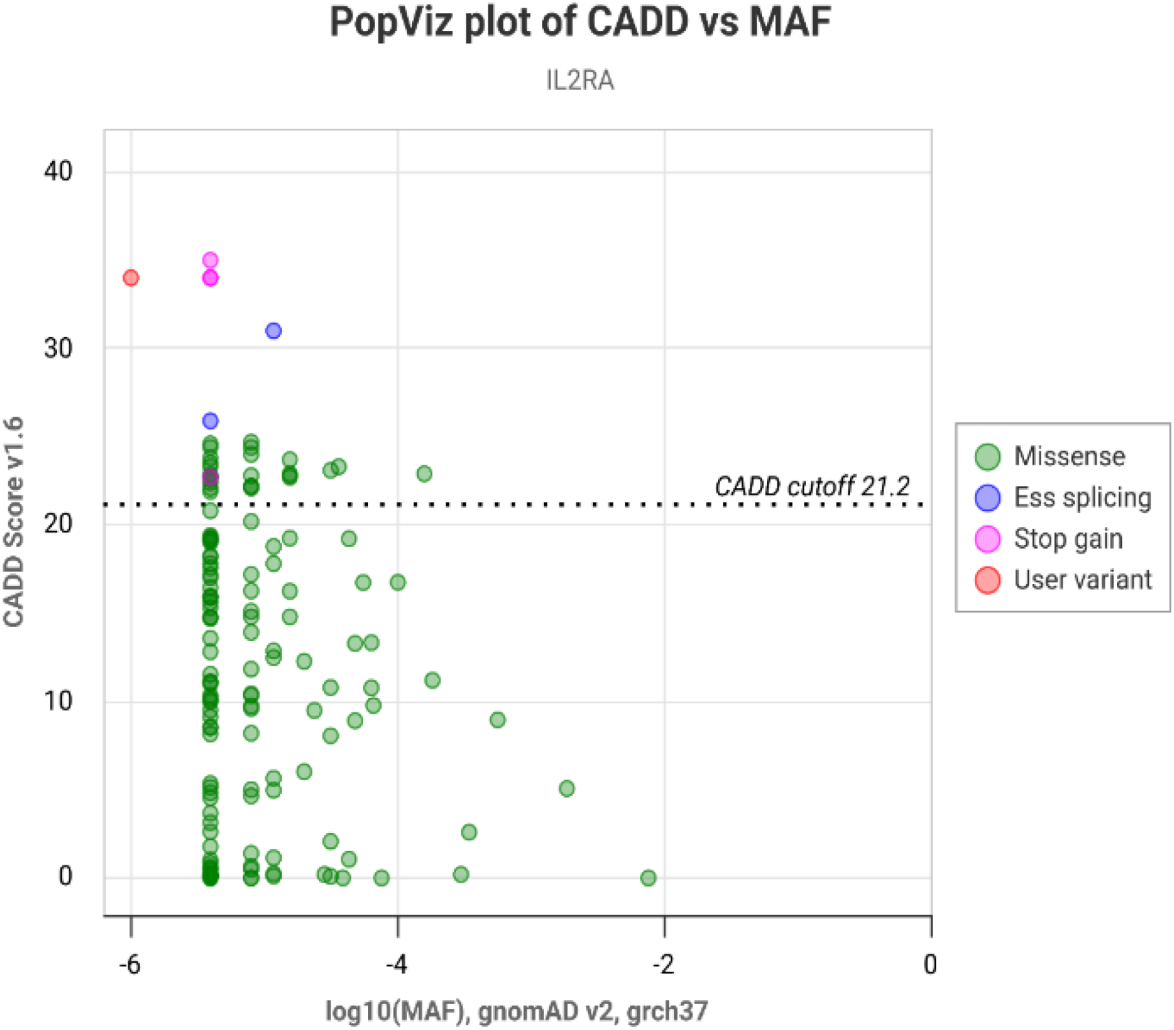
CADD vs. MAF plot of CD25 by PopViz. The vertical and horizontal axes show the combined annotation-dependent depletion (CADD) and the minor allele frequency (MAF) scores, respectively. The red dot is the c.65-2A>G variant and the other dots are the other variants. The log_81_ MAF is −6 and the CADD score is 34, which indicates that the new mutation c.65-2A>G is probably deleterious.

In Case 1, NGS revealed a homozygous splice-site variant in IL2RA, NM_000417.3: c.65-2A>G, located at the invariant acceptor site of intron 2, immediately upstream of exon 3 (**Figure 2B**). This change affects the canonical 3′ splice acceptor site and is predicted to abolish normal splicing of exon 3. The variant was absent from population databases (including gnomAD) and was not listed in ClinVar or other publicly available disease variant repositories at the time of analysis, indicating that it is extremely rare and previously unreported. In silico prediction tools uniformly supported a deleterious effect: the variant showed a high CADD score (34), a BayesDel_addAF score of 0.56, and splice-site prediction scores from dbscSNV (ADA = 1.00; RF = 0.93), together with an elevated Eigen score (0.85) (**Supplementary Table 1**). In PopViz, c.65-2A>G clustered among variants with high predicted deleteriousness and very low minor allele frequency (**Figure 4**). On the basis of these data, and given that loss-of-function of IL2RA is a known mechanism of disease, this variant was classified as likely pathogenic according to ACMG criteria (PVS1, PM2, PP3).

For Case 2, NGS read-depth analysis identified a homozygous multi-exon deletion in *IL2RA*, corresponding at the cDNA level to c.557_795-1625del. This deletion encompasses the 3′ part of exon 4 and all of exons 5–7, removing a substantial portion of the coding sequence that includes the Extracellular domain and transmembrane domains of CD25 (**Figure 2B**). Such a structural alteration is expected to produce a truncated, non-functional protein or lead to degradation of the transcript through nonsense-mediated decay, and therefore represents a clear loss-of-function allele. The deletion was not found in population databases and, to our knowledge, has not been previously reported. Because this is a large exon-level deletion, standard single-nucleotide in silico prediction scores (such as CADD or BayesDel) are not applicable; pathogenicity was therefore assessed on the basis of the predicted protein truncation, the essential domains affected, and its absence from controls. According to ACMG criteria, this variant was classified as pathogenic (PVS1, PM2).

Taken together with the complete absence of CD25 expression on CD4^+^ T cells and the IPEX-like clinical presentation, the identified biallelic *IL2RA* variants establish the diagnosis of IL-2Rα (CD25) deficiency in both affected children.

## 4 Discussion

We report two pediatric cases of complete IL-2Rα (CD25) deficiency, each caused by novel homozygous mutations, representing the first documented patients from Morocco and, to our knowledge, the North African region. These patients underscore key features of this syndrome and have implications for diagnosis and management in our context. CD25 deficiency results in a distinctive immunological phenotype that combines features of immunodeficiency with severe autoimmunity (10–18). Due to the absence of CD25, IL-2 signaling through the IL-2R is virtually absent in patient T cells, especially affecting regulatory T cells that require IL-2 for survival and function. As a consequence, patients are unable to maintain normal immune tolerance, leading to multi-organ autoimmunity, such as enteropathy, endocrinopathies, dermatitis, and cytopenias, early in life (12–14). At the same time, IL-2 is critical for effective immune responses; thus these patients also experience vulnerability to infections despite having normal or even expanded T-cell numbers (15, 16). The combination of recurrent infections (often opportunistic or chronic viral infections) with IPEX-like autoimmune disease is a hallmark of CD25 deficiency (17). Both of our patients exhibited this combination, with persistent viral infections and severe chronic diarrhea and endocrine autoimmunity akin to IPEX syndrome. Indeed, CD25 deficiency has been described as an “IPEX-like” disorder in the literature (11), since the clinical presentation overlaps considerably with FOXP3-deficient IPEX, apart from the inheritance pattern (CD25 deficiency is autosomal recessive, affecting both genders equally, unlike X-linked IPEX). One distinguishing feature observed in prior CD25-deficient cases is extensive lymphoproliferation – for example, Sharfe et al. noted lymphocytic infiltrates in lung, liver, gut, and other tissues of their patient (10). Our patients also had significant hepatosplenomegaly and lymph node enlargement, consistent with lymphoproliferation. The immunopathology likely reflects uncontrolled activation and expansion of autoreactive T cells that lack regulation by Tregs (11, 12). In line with previous reports, we found that both patients had no detectable CD25 on their T cells, confirming the diagnosis at a protein level. This was also true in all other published cases of IL2RA mutations. To date, all reported patients showed absent CD25 expression on flow cytometry (**Table 3**) (10–18). This immunophenotypic hallmark can be invaluable for diagnosis: in an infant or young child with unexplained polyautoimmunity and infections, flow cytometric analysis of CD25 on T cells can provide an immediate clue to IL2RA deficiency, even before genetic results are available.

**Table 3:** Reported cases of CD25 deficiency: a comparative genotype-phenotype summary.

Case (year)	Country/Demographics	Genetic mutations (IL2RA)	CD25 expression	Clinical & immunological features	Reference
Our patient 1	Morocco (M, infant, consanguineous parents)	Homozygous splice-site (c.65-2A>G) located at the invariant acceptor site of intron 2, immediately upstream of exon 3	Absent – No CD25 surface expression on T cells	Frequent respiratory infections, multiple pneumonias, dysentery; persistent viral infections, Autoimmune enteropathy (celiac disease), autoimmune hepatitis, eczema	-
Our patient 2	Morocco (M, infant, consanguineous parents)	Homozygous multi-exon deletion in *IL2RA deletion of exons 5–7 and part of exon 4 (c.557_795-1625del)*	Absent – No CD25 surface expression on T cells (null mutation).	Chronic respiratory infections and diarrheal illness, Autoimmune enteropathy (celiac disease), hypothyroidism, dermatitis (eczema)	-
Sharfe et al., 1997	Canada/Israel (M, infant, consanguineous parents)	Homozygous 4-bp deletion in exon 1 (c.60_64del4) → frameshift & premature stop (p.F21fs*46).	Absent – No CD25 surface expression on T cells (null mutation).	SCID-like with autoimmunity: Onset at 6 months; recurrent bacterial, viral (CMV) and fungal infections; chronic diarrhea, failure to thrive, lymphadenopathy, hepatosplenomegaly. Also developed IPEX-like autoimmunity (e.g. autoimmune biliary cirrhosis) despite low T cell counts. Poor T-cell proliferative responses; survived infancy with supportive care.	Sharfe et al., 1997 (PNAS) (10)
Caudy et al., 2007	USA (M, infant, non-consanguineous)	Compound heterozygous: one nonsense (exon 3, c.301C>T → p.R101*) and one frameshift (c.693insA → p.Y232fs*)?.	Absent – No CD25 on T cells; activated T cells failed to upregulate CD25.	Early-onset IPEX-like syndrome: Onset at 6 weeks with autoimmune enteropathy (villous atrophy) and type-1 diabetes. Developed multiple autoimmune cytopenias (AIHA, neutropenia), eczema, chronic diarrhea, and recurrent infections (severe CMV pneumonitis, etc.). Defective IL-10 production by CD4 T cells noted. Poor mitogen responses; combined immunodeficiency with immune dysregulation.	Caudy et al., 2007 (JACI) (11)
Goudy et al., 2013	Italy (F, infant, consanguineous)	Homozygous missense in exon 4 (c.497G>A) → p.S166N; IL2RA null mutation (no functional protein).	Absent – CD25 not expressed on surface (misfolded protein).	Severe autoimmune & lymphoproliferative syndrome: Onset at 1 month with intractable enteropathy (severe autoimmune enteritis), erythroderma, failure to thrive. Developed alopecia universalis by age 5. Course complicated by chronic CMV infection and EBV-driven lymphoproliferation. Regulatory T cells present but with impaired function; pronounced CD8^+^ T-cell expansion. Eventually treated with HSCT.	Goudy et al., 2013 (Clin Immunol) (12)
Bezrodnik et al., 2014	Argentina (F, infant)	Homozygous missense in exon 2 (c.122A>C) → p.Y41S.	Absent – No CD25 detected on CD4 T cells; extremely low Tregs (CD25−FOXP3+).	Combined immunodef. with lung involvement: Onset at 6 days old with diffuse eczema, enteropathy and recurrent infections. Later developed follicular bronchiolitis (chronic inflammatory lung disease with lymphocytic hyperplasia). Also had alopecia by age 5 and chronic diarrhea. Hypergammaglobulinemia with absent IgG4. Managed with immunosuppression (steroids, rapamycin, IVIG) pending HSCT.	Bezrodnik et al., 2014 (Clin Exp Immunol) (15)
Al Sukaiti et al., 2014	Oman (M, infant, consanguineous)	Homozygous missense (c.418T>C) in IL2RA (novel) → likely p.Y140H; mutation in exon 4 disrupting receptor extracellular domain.	Absent – No functional CD25 on T cells (confirmed by flow cytometry).	SCID phenotype with autoimmunity: Early infancy onset with severe pneumonia and pulmonary hemorrhage requiring ventilation. Suffered severe viral (CMV) and bacterial infections and autoimmune enteropathy. This Omani case uniquely had life-threatening pulmonary hemorrhage as a presentation. Treated with antimicrobials and supportive care; outcome improved with eventual HSCT.	Al Sukaiti et al., 2014 (LymphoSign J) (16)
Gonçalves et al., 2017	Brazil (M, 17 y/o adolescent)	Homozygous missense in exon 1 (c.100C>T) → p.A34T (novel variant).	Absent – No CD25 (flow cytometry confirmed IL-2Rα deficiency).	Atypical late-onset case: Lifelong history of recurrent pneumonias, skin infections (furunculosis), allergic rhinitis/asthma, and chronic diarrhea since childhood. Developed *celiac-like* disease (gluten-sensitive enteropathy) with positive anti-gliadin but normal biopsy on diet. Lung biopsy showed follicular bronchiolitis with organizing pneumonia. Low IgM and NK-cell counts; poor delayed-type hypersensitivity responses. Managed with azithromycin prophylaxis and IVIG (no transplant reported).	Gonçalves et al., 2017 (JACI abstract) (30)
Baş et al., 2018	Turkey (F, 12 y/o child)	Homozygous IL2RA mutation (specific variant not stated; known pathogenic) – likely missense.	Absent – No CD25 expression (consistent with prior cases).	Ocular autoimmunity prominence: Presented with severe ocular surface disease – intractable dry eye syndrome and keratitis due to T-cell infiltration. Also had history of eczema and recurrent infections (consistent with CD25 deficiency). This case highlighted significant meibomian gland dysfunction requiring topical immunosuppressants (cyclosporine). Systemic features mirrored IPEX-like immune dysregulation (managed by immunologists in Turkey).	Baş et al., 2018 (Eye & Contact Lens) (18)
Vignoli et al., 2019	Italy (M, 1 y/o infant)	Novel IL2RA conformational mutation (exon 4 missense) causing protein misfolding and no surface expression. (Likely compound heterozygous, one allele p.Cys**→**Arg; exact variant not in abstract.)	Absent – Total lack of CD25 on cell surface (flow cytometry).	Fulminant IPEX-like in infancy: Onset in neonatal period with enteropathy, eczema, insulin-dependent diabetes, and failure to thrive. Also had severe viral and bacterial infections (due to combined immunodeficiency). Early genetic diagnosis allowed HSCT by age 1, which completely cured the disease (resolution of autoimmunity and infections). This underscores the benefit of early transplant in CD25 deficiency.	Vignoli et al., 2019 (Clin Immunol) (17)
N Lai et al., 2020	China (M, 8 y/o child)	Compound heterozygous IL2RA mutations (two novel variants: one splice-site, one missense). Both alleles inherited from carrier parents.	Low/Absent – Essentially no functional CD25; very low Treg frequency.	Immune dysregulation with HLH-like features: Presented with recurrent high-grade fevers, hepatosplenomegaly, cytopenias and refractory EBV+ hemophagocytic lymphohistiocytosis. Also had chronic diarrhea and eczema. Standard treatments failed. Notably, rapamycin therapy was introduced, leading to marked clinical improvement and control of immune activation. This case demonstrated mTOR inhibition as a potential therapy in IL2RA deficiency. (Patient proceeding to HSCT.)	N Lai et al., 2020 (JACI In Practice) (27)
Alaifan et al., 2022	Saudi Arabia (M, 12 y/o boy)	Homozygous nonsense in exon 4 (c.504C>A) → p.C168* (truncation of receptor by 272 aa). Likely null allele (no IL2RA protein).	Absent – No CD25 expression; null mutation causes complete IL-2Rα deficiency.	Chronic immune dysregulation: Symptom onset at 7 months with protracted diarrhea, failure to thrive, eczematous dermatitis. Developed granulomatous hepatitis (elevated liver enzymes, biopsy with ill-defined granulomas) – *first CD25-deficient patient reported with granulomas in liver*. Also had mild villous atrophy on duodenal biopsy and recurrent infections (UTI, etc.). No evidence of TB or other causes for granulomas. Managed conservatively; spontaneous improvement in liver inflammation, awaiting HSCT.	Alaifan et al., 2022 (Ther. Adv. Chronic Dis.) (13)
Korula et al., 2023	India (M, 4 mo infant, consanguineous)	Homozygous missense IL2RA mutation (variant reported in db but not previously published in a case). (Likely same p.S166N or p.Y41S as earlier cases – exact not specified.)	Absent – Flow cytometry confirmed lack of CD25 on T cells; FoxP3^+^ Tregs present but CD25^-^	Neonatal diabetes & enteropathy: Presented at 2 months with neonatal-onset diabetes mellitus (NDM) along with protracted diarrhea and failure to thrive. Work-up revealed IL2RA defect; infant was GAD-antibody positive (unusual association). Due to immune dysregulation and insulin-requiring diabetes, HSCT was recommended. (This case is reported as the first IL2RA deficiency from India; unfortunately the family was lost to follow-up before transplant).	Korula et al., 2023 (Case Report – India) (28)

The genetic findings in our patients expand the known mutational spectrum of *IL2RA*. Prior to this report, approximately 15 cases from various ethnic backgrounds had been documented in the literature (10–18). The majority of reported mutations are either missense substitutions that disrupt protein expression/folding or nonsense/frameshift mutations leading to truncated proteins (10–15, 17). For example, Sharfe et al. reported a frameshift (4-bp deletion) in *IL2RA* that produced a nonfunctional receptor (10). Vignoli et al. described a conformational missense mutation that prevented IL-2Rα from reaching the cell surface (17). Our Case 1 has a splice-site mutation (c.65-2A>G) which is predicted to cause abnormal mRNA splicing and likely results in an out-of-frame transcript or nonsense-mediated decay. Notably, this splice mutation is novel and had not been recorded in any variant databases, emphasizing the uniqueness of each family’s mutation in this ultra-rare disease. Our Case 2 harbors a large deletion spanning multiple exons of *IL2RA*. The multi-exon deletion c.557_795-1625del identified in patient 2 represents, to our knowledge, the first reported structural null allele of IL2RA. This deletion results in the complete loss of several essential exons, accounting for the observed absence of CD25 expression. Its identification highlights the importance of actively investigating copy number variations in patients with IPEX-like phenotypes, particularly when conventional sequencing fails to detect point mutations. This finding underscores the role of multi-exon deletions as an underlying genetic mechanism of IL2RA deficiency and enhances our understanding of CD25 loss in these patients. Large deletions can be challenging to detect by standard gene panel sequencing, but in our targeted NGS approach we were able to infer the deletion from read-depth analysis. These two mutations underscore that *IL2RA* pathogenic variants can be diverse (point mutations, indels, splice defects, large deletions), and comprehensive genetic testing is often required to identify them.

Clinically, both patients reinforce certain patterns noted in CD25 deficiency. First, the onset of disease is in early infancy. Patient 1 and patient 2 both showed symptoms within the first year of life. This is consistent with the notion that IL-2Rα is critical from the neonatal period for immune regulation. Second, consanguinity was present in both families. Given the autosomal recessive inheritance, CD25 deficiency is more likely to appear in settings of parental consanguinity or endogamous communities. This is an important epidemiological clue; indeed, most previously reported cases were from consanguineous families, including reports from communities in the Middle East and South Asia (16, 17). Our report highlights that this disorder, while exceedingly rare, can also occur in North Africa, and underscores the need to consider *IL2RA* mutations in patients from this region presenting with IPEX-like features. Third, while both our patients had multiple autoimmune diseases, the exact spectrum can vary. Case 1 had severe autoimmune hepatitis, whereas Case 2 had autoimmune thyroiditis. In the literature, autoimmune enteropathy is the most common feature (16), and both of our patients indeed had enteropathy, with biopsy-proven celiac-like disease. Other reported autoimmune manifestations include type 1 diabetes mellitus, which can be an early presenting feature in up to ~60% of cases, autoimmune skin conditions (eczema or psoriasis), and autoimmune cytopenias (11, 16, 27, 28). Neither of our patients has developed diabetes to date, although they remain at risk given the breakdown of tolerance (regular monitoring is in place). Unlike FOXP3 deficiency, IL2RA deficiency does not involve mutations in FOXP3 but results from impaired IL-2 signaling required to sustain FOXP3 expression in Tregs (11, 12, 16). Despite some clinical differences, including occasionally more pronounced lymphoproliferation, the strong phenotypic overlap supports classifying CD25 deficiency among IPEX-like syndromes, alongside STAT5b, CTLA4, and LRBA deficiencies (17). Although hyperleukocytosis and elevated IgG levels were observed in both patients, these findings alone do not constitute a specific biological profile that would allow a definitive diagnosis of CD25 deficiency. In contrast, the absence of CD25^+^ cells represents the key element in the diagnostic reasoning.

From a management standpoint, the definitive treatment for CD25 deficiency is hematopoietic stem cell transplantation (HSCT). Restoring a functional immune system via HSCT, ideally from an HLA-matched sibling or suitable donor, has been shown to cure the immune dysregulation in at least one reported case (16). Al-Sukaiti et al. documented an infant with CD25 deficiency who underwent a successful bone marrow transplant, resulting in resolution of his autoimmune diabetes and other manifestations (16). HSCT provides donor-derived T cells that express CD25, thereby re-establishing IL-2 responsive regulatory T cells and normalizing immune homeostasis. Both of our patients are being evaluated for potential HSCT while receiving supportive therapy. In the interim, meticulous multidisciplinary care is required: management includes aggressive treatment of infections (prophylactic antibiotics, antifungals and antivirals as needed) and immunosuppressive therapy to control autoimmunity, for example, Case 2’s hypothyroidism is managed with hormone replacement and her enteropathy with immunosuppressants. The use of therapies like rapamycin (sirolimus) that expand regulatory T cells by exploiting IL-2 signaling could be considered, although paradoxically in CD25 deficiency the Tregs cannot respond normally to IL-2 (27). Low-dose IL-2 therapy, which has shown promise in boosting Tregs in some autoimmune conditions (29), would presumably be ineffective in CD25 absence. Therefore, immune reconstitution by transplantation remains the only curative option. Early diagnosis is essential to enable timely HSCT before irreversible organ damage from ongoing autoimmunity. Our cases highlight that CD25 deficiency should be considered in infants with unexplained multisystem autoimmunity particularly enteropathy and endocrinopathy associated with recurrent infections, especially in the context of consanguinity or affected siblings. Flow cytometric assessment of CD25 expression on T cells provides a simple initial screening tool.

These two Moroccan cases expand the geographic and genetic spectrum of IL2RA deficiency. They are, to the best of our knowledge, the first reported patients from Morocco. Prior to this, fewer than 20 cases had been recorded globally, with the largest series noting only about eight families worldwide as of a few years ago (17). Our report adds novel mutations to the literature, including a unique large deletion, and reinforces the genotype-phenotype correlation that any null mutation in *IL2RA* will lead to the characteristic severe autoimmune immunodeficiency syndrome. The rarity of CD25 deficiency means it is often not immediately recognized. Many patients, like the brother of Case 2, may succumb before a diagnosis is made. Increasing awareness among immunologists and pediatricians is therefore vital. In settings where infantile IPEX-like symptoms are present but FOXP3 is normal (or the patient is female, ruling out X-linked IPEX), testing for *IL2RA* mutations is indicated.

This study has some limitations that should be acknowledged. First, it is based on two patients from a single center, which does not allow firm conclusions about the prevalence, full phenotypic spectrum or long-term prognosis of IL2RA deficiency, particularly in North African populations. Second, although we documented a complete absence of CD25 surface expression and used several in silico tools to support the pathogenicity of the identified variants, we did not perform functional assays of IL-2 signaling (such as STAT5 phosphorylation or regulatory T-cell suppressive function) or transcript-level studies to directly confirm the splicing consequences of the c.65-2A>G variant and the impact of the multi-exon deletion at RNA and protein level. Third, genetic testing was restricted to the two index cases, and we were therefore unable to formally confirm carrier status in all relatives or to document the molecular defect in the deceased sibling of Case 2. Finally, follow-up remains relatively short, especially for the younger child, so later-onset complications cannot be excluded. These limitations do not weaken the diagnostic attribution of IL-2Rα deficiency in our patients, but they highlight the need for additional functional work and longitudinal data in larger cohorts.

## 5 Conclusion

We report the first two molecularly confirmed cases of IL2RA (CD25) deficiency in Morocco, each due to a novel homozygous variant, including a previously undescribed multi-exonic deletion. Both children developed disease in early life with recurrent infections, severe enteropathy and multi-organ autoimmunity, giving an IPEX-like picture typical of complete CD25 loss. The combination of absent CD25 expression on CD4^+^ T cells and biallelic IL2RA variants allowed a precise diagnosis in both cases. These observations extend the geographic and genetic spectrum of IL2RA deficiency and underline that this disorder should be considered in infants and children from consanguineous families who present with unexplained polyautoimmunity and infections, even outside previously described populations. Simple flow cytometric assessment of CD25 on T cells provides a rapid and accessible screening tool, while early genetic confirmation is essential to guide timely hematopoietic stem cell transplantation and genetic counselling. Larger collaborative series with detailed functional studies will be needed to refine genotype–phenotype correlations and to optimize management strategies for this ultra-rare but life-threatening inborn error of immunity.

## Data Availability

Datasets are available on request: The raw data supporting the conclusions of this article will be made available by the authors, without undue reservation.
