# Supplementary material for: Case Report: IL2RA (CD25) deficiency: first reported cases in Morocco
Source: Front Immunol. 2026 Jan 30;17:1753561. doi: 10.3389/fimmu.2026.1753561 (PMC12901503; doi:10.3389/fimmu.2026.1753561)
Supplement: Supplementary file 1 [file Table1.docx]

**Supplementary Table 1**. In silico deleteriousness prediction scores for IL2RA c.65-2A>G (Case 1).

| Prediction Tool | Classification | Score* |
| --- | --- | --- |
| BayesDel_addAF | Pathogenic | 0.56 |
| CADD | Pathogenic | 34 |
| dbscSNV (ADA) | Pathogenic | 1.00 |
| dbscSNV (RF) | Pathogenic | 0.93 |
| Eigen | Pathogenic | 0.85 |

**All in silico predictions were obtained using GeneBe and standard variant annotation tools. Higher scores in these algorithms indicate increased likelihood of pathogenicity.*
